# Supplementary material for: Saccharomyces boulardii (CNCM I-745) improves intestinal damage in sepsis by remodeling intestinal flora structure
Source: Front Cell Infect Microbiol. 2025 Jul 31;15:1602792. doi: 10.3389/fcimb.2025.1602792 (PMC12350329; doi:10.3389/fcimb.2025.1602792)
Supplement: Supplementary file 1 [file DataSheet1.zip › summary/summary/7_Differential_analysis/1_diff_abundance/3_Class/Group.BW4_vs_AW4/Group.BW4_vs_AW4_diff_barplot.pdf]

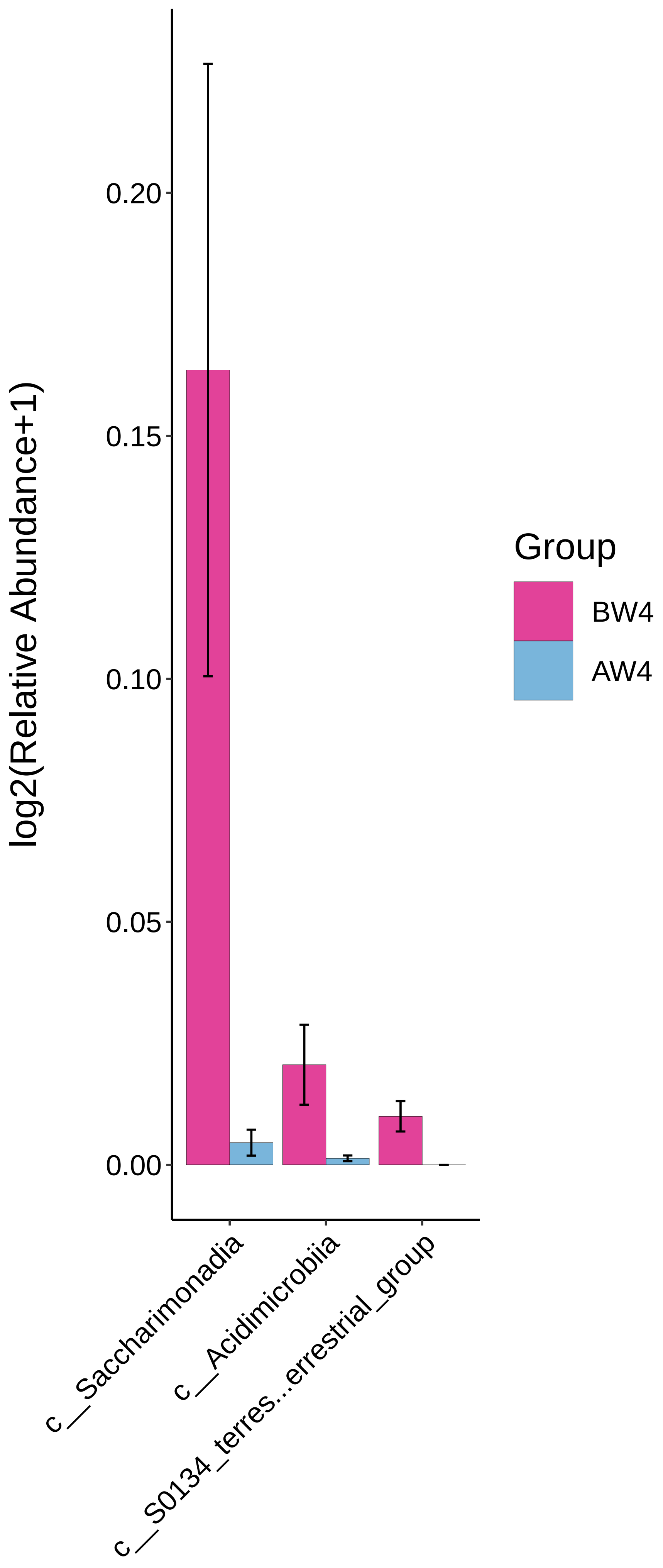

log2(Relative Abundance+1)
0.20
0.15
0.10
0.05
0.00
c__Saccharimonadia
c__Acidimicrobiia
c__S0134_terres...errestrial_group
Group
BW4
AW4
